# Insulin resistance in ischemic stroke: Mechanisms and therapeutic approaches

**DOI:** 10.3389/fendo.2022.1092431

**Published:** 2022-12-15

**Authors:** Peng-Fei Ding, Hua-Sheng Zhang, Jie Wang, Yong-Yue Gao, Jian-Nan Mao, Chun-Hua Hang, Wei Li

**Affiliations:** ^1^ Department of Neurosurgery, Nanjing Drum Tower Hospital Clinical College of Nanjing Medical University, Nanjing, China; ^2^ Department of Neurosurgery, Nanjing Drum Tower Hospital, The Affiliated Hospital of Nanjing University Medical School, Nanjing, China

**Keywords:** ischemic stroke, insulin resistance, atherosclerosis, embolism, therapeutic approaches

## Abstract

The pathological condition of insulin resistance prevents the neuroprotective effects of insulin. Numerous studies have demonstrated that insulin resistance, as an independent risk factor for ischemic stroke, accelerates the formation of thrombosis and promotes the development of atherosclerosis, both of which are major mechanisms of ischemic stroke. Additionally, insulin resistance negatively affects the prognosis of patients with ischemic stroke regardless of whether the patient has diabetes, but the mechanisms are not well studied. We explored the association between insulin resistance and the primary mechanisms of brain injury in ischemic stroke (inflammation, oxidative stress, and neuronal damage), looking for potential causes of poor prognosis in patients with ischemic stroke due to insulin resistance. Furthermore, we summarize insulin resistance therapeutic approaches to propose new therapeutic directions for clinically improving prognosis in patients with ischemic stroke.

## Introduction

Stroke incidence and patient prognosis have not changed significantly over the past few decades, despite significant advances in clinical interventions aimed at reducing stroke risk factors like hypertension, smoking, and diabetes. Stroke remains the second-leading cause of disability and death globally (1). Since energy metabolism is a prerequisite for life activity, many studies have examined disorders of energy metabolism in brain tissue, particularly insulin. Insulin protects brain tissue development by preventing ischemia, oxidative stress, and apoptosis-induced brain tissue damage, regulating cholesterol metabolism in neurons and astrocytes. Insulin also can effectively alleviate cognitive dysfunction caused by Alzheimer’s disease (2). Insulin resistance (IR) has long been linked to ischemic stroke, which makes up 87% of strokes and is increasing (3). IR, which is present in the majority of type II diabetes (T2D) patients, promotes the development of ischemic stroke and is associated with a poor prognosis (4, 5). One of the best-known effects of IR is the presence of hyperglycemia, which may negatively affect brain function through various mechanisms (6). This review evaluates studies on IR as an independent risk factor for ischemic stroke, with a particular focus on the mechanisms and therapy associated with IR and ischemic stroke. Firstly, we discuss the relationship between IR and the risk factors for ischemic stroke (hypertension, hyperlipidemia, etc.). Secondly, we discuss the potential causes of IR leading to poor outcomes in ischemic stroke patients. Lastly, we synthesize the research on IR inhibitors and ischemic stroke currently available and look ahead to a day when reducing IR may be a useful strategy for both preventing and treating ischemic stroke.

### Normal brain insulin signaling and IR

Insulin is a well-known hormone secreted by pancreatic β-cells, which regulates peripheral glucose metabolism. Insulin signaling from the central nervous system (CNS) regulates energy balance *via* complex mechanisms (7). The concentration of insulin in cerebrospinal fluid is correlated with the concentration in circulating plasma. Although it is still debated whether insulin can be produced in the CNS, circulating insulin can enter brain tissue *via* the (BBB) (8).

Neurons are the ultimate beneficiaries of brain tissue glucose uptake, consuming 85% of the energy needed by brain tissue (9). Brain tissue accounts for only about 2% of an adult’s body weight but consumes about 20% of total body energy, and glucose metabolism produces even more than 95% of the ATP required by brain tissue, making glucose metabolism in brain tissue especially important (10). Glucose transporter 3 (GLUT3) and glucose transporter 4 (GLUT4) are found on the neuronal cell membrane, with GLUT4 serving as a critical determinant of glucose homeostasis and being highly dependent on insulin (11, 12). Under insulin stimulation, GLUT4 translocation from the cytoplasm to the cell membrane in neurons in the hippocampus and cortex has a glucose-promoting transport effect (13). Insulin not only maintains the balance of energy metabolism in brain tissue but also stimulates neurite outgrowth, modulates catecholamine release and uptake, regulates the expression and localization of N-methyl-D-aspartate (NMDA), α-amino-3-hydroxy-5-methyl-4-isoxazolepropionic acid (AMPA) and γ-aminobutyric acid (GABA) receptors, and modulates synaptic plasticity to enhance neuronal survival by inhibiting apoptosis (14).

**Figure 1:**
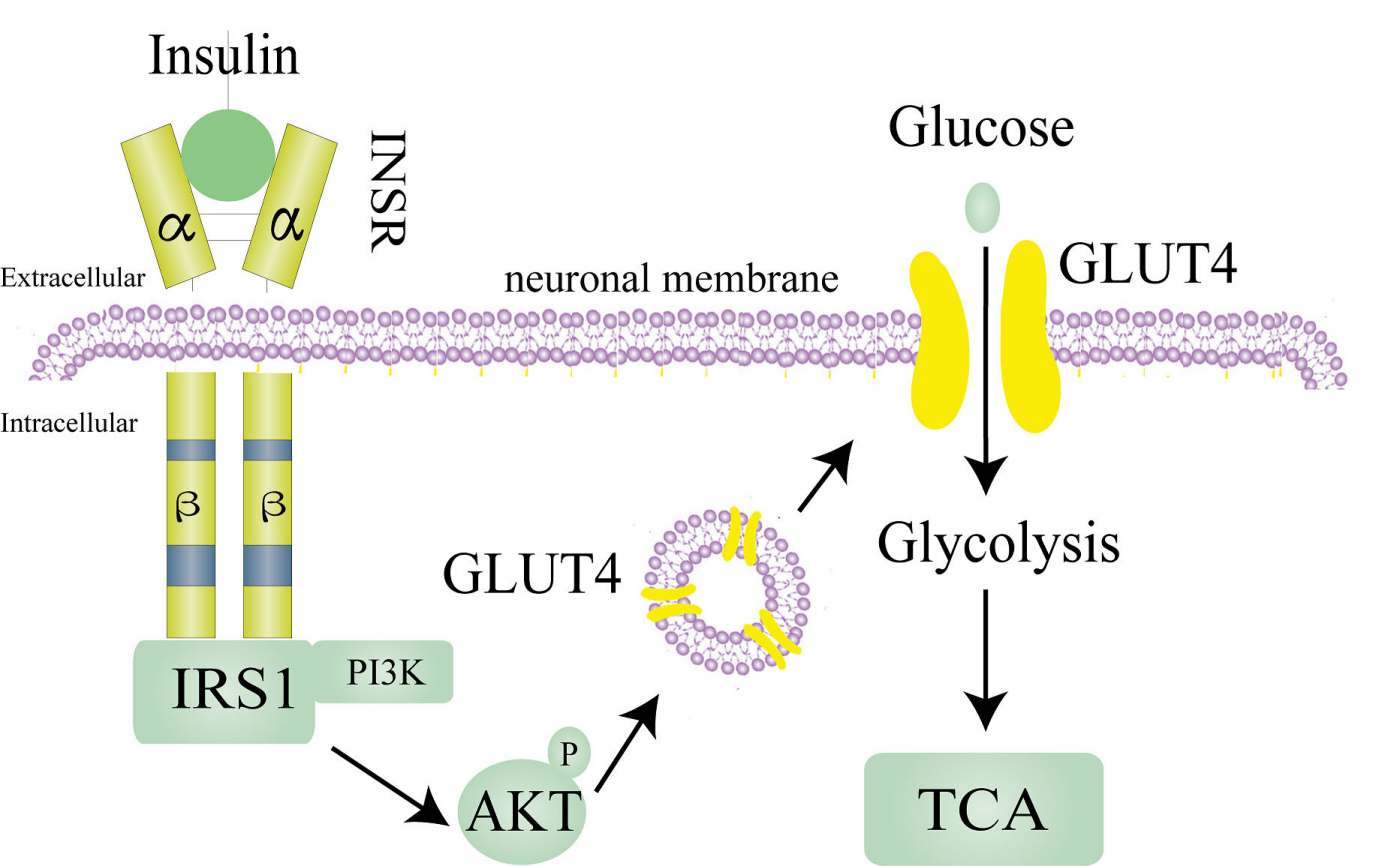
Normal insulin pathway in the neuron.

Insulin needs to bind to insulin receptors (INSR) on plasma membranes to exert its known biological effects. Insulin receptor, which consists of two extracellular α subunits and β subunits, are found in the brain and on most cells. In brain tissue, insulin receptors are mainly distributed in the hypothalamus, olfactory bulb, hippocampus, striatum, cerebral cortex, and cerebellum (14). Insulin first binds to the extracellular α subunits of insulin receptors, which induces the autophosphorylation of intracellular β subunits (15). INSR has two isoforms, A and B. Evidence shows that the brain only expresses the shorter form INSR-A rather than the full-length INSR-B, which differs from many peripheral tissues (16). The most classic INSR scaffold is the insulin receptor substrate (IRS) family, which has six isoforms (IRS1-6). IRS1 and IRS2 are assumed to mediate most of the metabolic effects of INSR activation (17). In brain tissue, insulin acts through IRS1, and IRS2 is strongly related to the activity of insulin-like growth factor 1(IGF-1) (16). By phosphorylating several IRS tyrosine residues, INSR attracts downstream signaling effectors to relay and enhance insulin responses. Tyrosine-phosphorylated IRS proteins active phosphoinositide 3-kinase (PI3K) and then phosphorylate AKT to exert the known physiological effects of insulin (**Figure 1**) **(** 17).

IR, as the name implies, is the failure of tissues to the normal response to insulin stimulation (18). IR appears earlier in brain tissue than in the periphery, suggesting that brain tissue is more vulnerable to IR, particularly in brain diseases such as ischemic stroke (14). There are four clinically accepted criteria for detecting the presence of IR in patients: (1) the gold standard assessment, homeostasis model assessment of IR (HOMA-IR); (2) oral glucose tolerance tests (OGTT); (3) C-peptide release test; and(4) triglyceride glucose (TyG) index, each with advantages and disadvantages (**Table 1**) **(** 19–22). In addition, the TyG index combined with obesity indices, including body mass index (BMI), waist circumference (WC), and waist height ratio (WHtR) were found to be more accurate than the TyG index alone. TyG-BMI had the best ability to detect IR and the best consistency with HOMA-IR among them (23). However, TyG-BMI, TyG-WC, and TyG-WHtR share the same advantages and disadvantages as the TyG index. At the molecular level, while abnormalities in INSR number, receptor kinase activity, and various receptors for insulin action can all lead to IR production in tissues but abnormal serine phosphorylation of IRS blocking the PI3K/AKT pathway is thought to be the main cause of IR (17, 24). As a result, the ratio of serine phosphorylation to total phosphorylated IRS can be used as a marker of IR in either the brain or peripheral tissues, with a higher percentage indicating increased IR (25).

**Table 1:** Four normal methods for assessment of IR in clinical.

Name	Method	advantage	Disadvantage
HOMA-IR	(Plasma insulin during fasting × plasma glucose during fasting)/22.5	Simple, minimally invasive, predicts fasting steady-state glucose and insulin levels	limitations because of significant heterogeneity of cut-off value and IR definitions; HOMA-IR may not be appropriate in patients with severely impaired or absent β-cell function
OGTT	After the overnight fast, fasting blood glucose levels were measured and then remeasured at the time point after drinking 75 g of glucose solution.	Simple, minimally invasive	Relatively crude measurement of glucose tolerance without measuring insulin sensitivity and insulin secretion components
C-peptide release test	After the overnight fast, fasting blood C-peptide levels were measured and then remeasured at the time point after drinking 75 g of glucose solution.	Simple, minimally invasive; Long half-life; It is not interfered with by insulin antibodies and can more accurately reflect the patient’s β-cell function.	Relatively crude; Needs to be analyzed in conjunction with blood glucose and insulin
TyG index	Fasting triglyceride [mg/dL] × fasting glucose [mg/dL]/2	Simple, minimally invasive; Suitable for clinical and epidemiological studies	limitations because of significant heterogeneity of cut-off value and IR definitions

HOMA-IR, Homeostasis model assessment of IR; OGTT, oral glucose tolerance tests; TyG index, triglyceride glucose index. IR, insulin resistance.

### How IR leads to ischemic stroke

**Figure 2:**
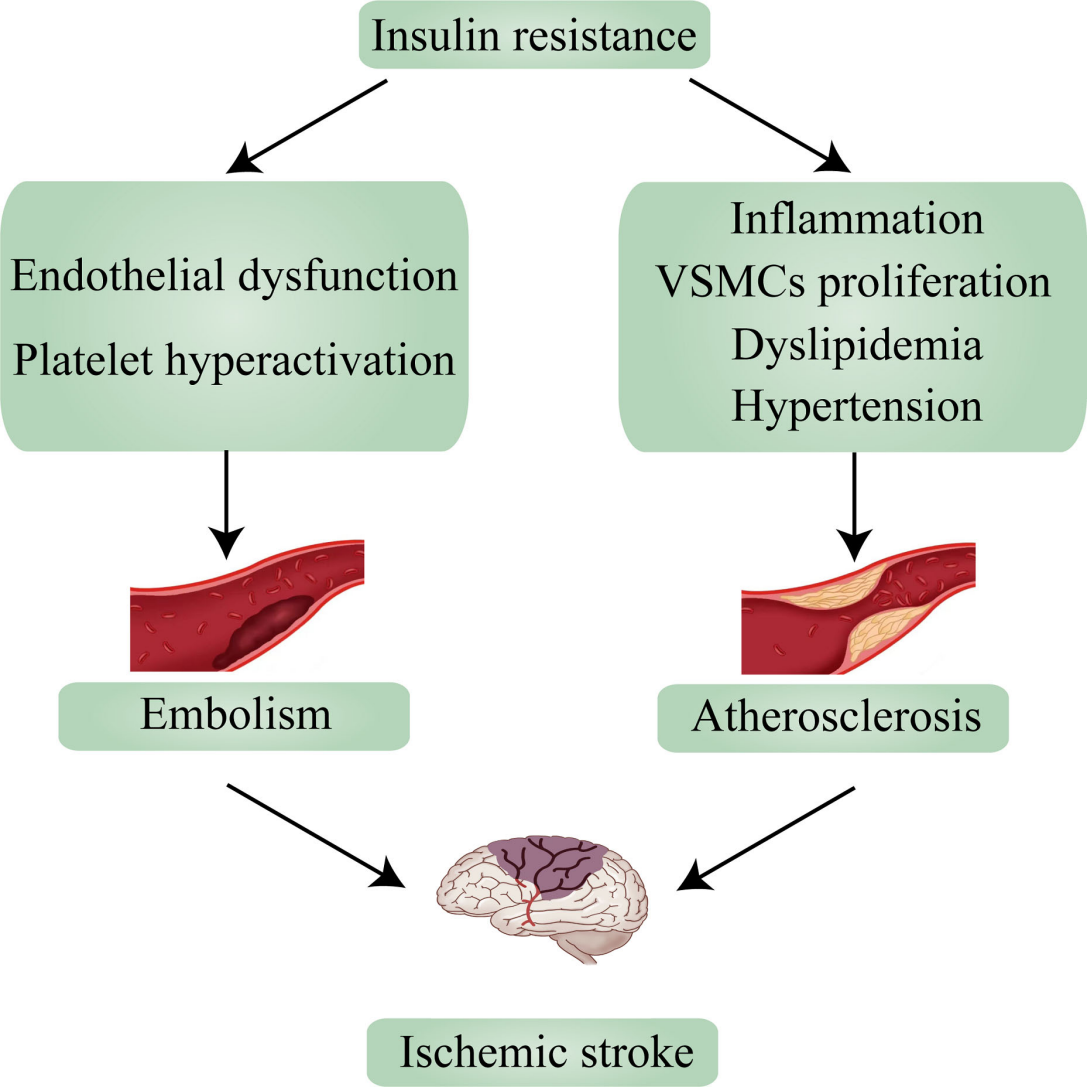
Insulin resistance leads to ischemic stroke *via* embolism and atherosclerosis. VSMCs, vascular smooth muscle cells.

Ischemic stroke mechanisms are classified as embolism, large vessel disease, and small vessel disease (26). In up to two-thirds of acute stroke patients, abnormalities in glucose regulation (of which diabetes is a manifestation) are observed, and because ischemic stroke accounts for 87% of strokes, there have been numerous studies on T2D and ischemic stroke (3, 27). According to research, ischemic stroke and T2D share many causative factors, including IR and IR-associated syndrome. An earlier study found that 50% of 72 nondiabetic patients with transient ischemic attack (TIA) or ischemic stroke had significant IR (28). And in non-diabetic ischemic stroke patients, the value of HOMA-IR ≥2. 5, suggesting the presence of IR, in more than 20% of cases (29). Numerous studies show that IR is a risk factor for ischemic stroke and can lead to the incidence of ischemic stroke (30). Based on past reviews and contemporary research, this section will explain the relationship between IR and the two main causes of ischemic stroke—embolism and atherosclerosis—and how it influences the occurrence and progression of ischemic stroke (**Figure 2**).

### IR promotes ischemic stroke through embolism

Ischemic stroke is most frequently caused by embolism. Most embolisms are blood clots that arise from the heart due to heart disease (cardiogenic embolism). Atrial fibrillation, heart valve disease, and myocardial infarction or cardiomyopathy brought on by excessive blood pressure are common cardiac disorders that cause stroke (26). Thrombosis is commonly considered to be a pathological hemostatic deviation caused by coagulation and platelet activation. The formation of an intravascular thrombus (clot) and vascular occlusion constitute thrombosis. Ischemic stroke occurs when dislodged clots move and block cerebral blood vessels (31). Interventions using antiplatelet agents to prevent platelet activation and thrombosis are known to reduce the incidence and severity of ischemic strokes. The metabolic environment of T2D includes IR, hyperglycemia, excessive release of free fatty acids, and other metabolic abnormalities affecting blood vessel walls as a result of a variety of events such as endothelial dysfunction and platelet hyperactivation. Compared to non-diabetes, T2D had a two-to four-fold higher risk of recurrent atherosclerotic thrombotic events and vascular complications. The activation of these events causes even more vasoconstriction and promotes thrombosis (32). IR, the key role of T2D, has also attracted the interest of researchers. A rising number of studies are focusing on the connection between IR and thrombosis. Researchers found that IR can impair endothelial cell function and enhance platelet adhesion, activation, and aggregation, resulting in the formation of thrombosis (33). Endothelial cells serve several functions, including pro- and anti-coagulation, which are balanced under normal conditions. When endothelial cell function is impaired, the risk of thrombosis increases (34). In IR, the PI3K pathway is impaired, resulting in decreased NO (vasodilator) production, whereas the MAPK pathway is activated, leading to increased ET-1 (vasoconstrictor) production and ultimately endothelial dysfunction (35). Excessive free fatty acids (FFA) release caused by IR and excess FFA may cause lipotoxicity, which leads to increased expression of coagulation tissue factors (e.g., PAI-1) *via* the same mechanism as glucotoxicity, resulting in a prothrombotic state (36). Furthermore, FFA can set off a vicious cycle by activating several pathways which increases the percentage of IRS1 serine phosphorylation (37). Chronic hyperglycemia and decreased serum paraoxonase/arylesterase 1 (PON-1) activity may impair high-density lipoprotein’s (HDL) anti-inflammatory capacity. Eventually, serum tumor necrosis factor-α (TNF-α), interleukin-6 (IL-6), and C-reactive protein (CRP), which respond to inflammatory levels, were eventually elevated, indicating a link between the inflammatory response caused by IR and endothelial dysfunction (38). In addition, IR, assessed by HOMA-IR, was associated with endothelial dysfunction in patients with chest pain and non-diabetic patients with normal myocardial perfusion (39).

Several studies have shown that IR promotes vascular occlusion and cardiovascular disease (CVD) development by influencing platelet adhesion and aggregation (32). Platelets’ major function in the body is to support primary hemostasis and intravascular blood flow. Platelet adhesion, aggregation, and release are the three steps in the platelet activation process. Adhesion to the subendothelial extracellular matrix occurs through the initial interaction of the matrix with specific receptors on the platelet, including the GP1b/V/IX complex bound to the Von Willebrand factor and the GPVI and αIIβ1 receptors bound to the collagen component of the extracellular matrix on the platelet surface. Firm adhesion leads to initial clot or thrombus formation, and activated platelets bound within the thrombus will begin to take up new platelets from the circulation *via* platelet-platelet interactions mediated by integrin receptor αIIbβ3. Platelets express two purinergic receptors, P2Y1 and P2Y12, which have been shown to play an important role in platelet activation, one of which (P2Y12) is a target for antiplatelet therapy (40). Treatment with clopidogrel and aspirin improves prognosis in patients with cerebral infarction without increasing the risk of moderate to severe bleeding (41). The effect of hyperinsulinemia on platelets is complex and varies between insulin-resistant patients and healthy individuals. In healthy individuals, insulin reduces platelet aggregation and the release of pro-aggregation agents by facilitating the transfer of magnesium to platelets (such as thromboxane B2) (42). Insulin naturally inhibits platelet hyperactivity. It makes platelets more sensitive to prostacyclin (PGI2) and increases endothelial cell production of PGI2 and nitric oxide (NO), allowing platelets to maintain normal function. Platelet hyperactivation favors macrovascular and microvascular events when IR occurs, which may explain why platelets adhere to the vascular endothelium more frequently in T2D patients than in healthy individuals (43). In addition, hyperglycemia may activate platelets through miR-144 and miR-223 to downregulate IRS1 and upregulate P2Y12 expression in the platelets *via* IRS1/PI3K/AKT pathways (44).

### IR facilitates ischemic stroke *via* atherosclerosis

Atherosclerosis is another common underlying cause of ischemic stroke (26). Atherosclerosis is a chronic inflammatory disease of large and medium-sized arteries that can lead to ischemic heart disease, stroke, and peripheral vascular disease collectively referred to as CVD (45). Increasing evidence has demonstrated that atherosclerosis is a major cause of ischemic stroke (46). Furthermore, there is strong evidence that IR or metabolic syndrome caused by IR contributes to the pathogenesis of ischemic stroke by promoting the formation of atherosclerotic and advanced plaques in the progression of atherosclerosis (47). According to epidemiological studies, hyperinsulinemia is an independent risk factor for atherosclerosis. Hyperinsulinemia caused by IR can exacerbate atherosclerosis by promoting vascular inflammation, vascular smooth muscle cells (VSMCs) development, a pathological cholesterol profile, hypertension, and immune cell recruitment to the endothelium (48). The relationship between inflammation and T2D can be traced back to the 1950s. At the molecular level, IR is promoted when macrophage polarization shifts from an alternative M2 (anti-inflammatory) activation state maintained by STAT6 and PPARs to a classical M1 (pro-inflammatory) activation state driven by NF-B, AP1. In short, inflammation promotes the development of IR (49). In contrast, researchers gave a 4-hour insulin intervention to healthy volunteers with normal glucose tolerance and no history of diabetes, took biopsies of their lateral femoral muscles, and discovered noticeably elevated levels of related inflammatory genes (50). We are aware of a mutual relationship between IR and inflammation. With both protective and pathogenic roles, monocyte-macrophage lineage cells and VSMCs are two key participants in the atherosclerotic process. Proliferation of VSMCs promotes plaque growth while also forming the atheroma’s fibrous cap (51). Hyperinsulinemia, which is caused by IR, is a potential growth factor that promotes its growth through the MAPK pathway, which catalyzes the phosphorylation of transcription factors that stimulate VSMCs growth, proliferation, and differentiation (52). It has been demonstrated that IR-related inflammation can also promote the development of VSMCs (53). C-peptide, produced by the cleavage of proinsulin in the β-cell. It is secreted equimolarly with the other cleavage product, insulin (54). C-peptide study extends to our knowledge of the mechanisms that promote VSMCs proliferation in hyperinsulinemia conditions (55). PI3K/AKT and ERK1/2-MAPK are thought to be critical signaling pathways controlling VSMCs. Through the activation of the protein tyrosine kinase Src, which can function as an intermediary in signaling networks that link G-protein-coupled receptors with downstream signaling cascades like the PI3K/Akt and the Ras/MAPK pathway, C-peptide can stimulate the growth of VSMCs (56, 57).

Atherosclerosis develops as a result of a disturbed cholesterol homeostasis balance (58). Numerous studies have shown that IR leads to disorders of lipid metabolism (59). In comparison to the normal metabolic state, insulin resistance promotes excess *de novo* lipogenesis as well as the production and secretion of very-low-density lipoprotein (VLDL) (60). Additionally, IR can accelerate the production of connective tissue in blood vessel walls and the aggregation of LDL cholesterol into arterial smooth muscle, both of which directly accelerate the development of atherosclerosis and, eventually, the occurrence of an ischemic stroke (47).

Hypertension is frequently regarded as a major cause of hemorrhagic stroke, as well as a risk factor for ischemic stroke (3). IR and hypertension are both independent risk factors for CVD, and a growing number of studies are beginning to recognize the link between these two diseases that promote the formation and progression of atherosclerosis, which together lead to CVD (61). Despite the fact that a short period of insulin stimulation did not significantly increase blood pressure in non-diabetic subjects, the researchers acknowledge that IR is directly correlated with the severity of hypertension (62). In a subsequent study, the researchers prolonged the insulin intervention and discovered that chronic insulin administration can significantly raise the blood pressure of lean rats (63). Leptin is crucial for controlling body weight, plays a role in the development of the IR syndrome, and is associated with cardiovascular disease. Increased sympathetic activity mediated by leptin may cause short- and long-term alterations in blood pressure through central and peripheral effects, which may help to explain how IR affects blood pressure (64). Although studies in humans appear controversial, the role of insulin in promoting antidiuretic effects and stimulating sympathetic nervous system activation has been confirmed as a potential mechanism by which insulin may elevate blood pressure (65). Furthermore, animal studies have shown that long-term insulin administration accelerates the development of atherosclerosis (47).

## How IR affects the progression of ischemic stroke

Stroke prevention and treatment are divided into two parts. Primary prevention includes modifying one’s lifestyle and treating risk factors such as hypertension, diabetes mellitus, etc. Secondary prevention includes surgical intervention and treatment of IR, etc. (66). In clinical studies, researchers have found that IR is independently associated with poor functional outcomes after acute ischemic stroke, regardless of whether the patient has T2D (5, 67). However, at the mechanistic level, it has not been well studied how IR negatively affects the prognosis of patients with ischemic stroke. Starting with the potential damage caused by IR, we summarize the potential mechanisms by which IR contributes to the poor prognosis of ischemic stroke patients in this section.

### IR and inflammation

The inflammatory response is believed to play a crucial part in the cerebral damage caused by an ischemic stroke (68). Microglia are important immune cells in brain tissue. Brain microglia are activated in response to ischemia. On the one hand, activated microglia secrete pro-inflammatory factors such as TNF-α and interleukin-1β (IL-1β), which cause cellular damage; on the other hand, activated microglia have phagocytic and major histocompatibility complex (MHC) class II-restricted antigen-presenting properties, which help remove dead tissue and debris after ischemia. Furthermore, activated microglia can promote the production of neurotrophic growth factors such as brain-derived neurotrophic factor (BDNF) (69, 70). This is related to the state of microglia polarization. Microglia polarization refers to the development of a classically activated (M1, pro-inflammatory) or alternatively activated (M2, anti-inflammatory) phenotype of activated microglia (71). M1 and M2 are in a dynamic equilibrium under normal physiological conditions, but in ischemic stroke, M2 is converted to M1 (72). Severe ischemic injury accompanied by a pro-inflammatory environment can produce and release large amounts of inflammatory factors causing ischemic damage to brain tissue, among which IL-6 and TNF-α can also lead to an increase in neutrophils in circulating dead brain tissue. Increased neutrophil counts not only correlate with infarct size but also can disrupt the BBB (73). Obesity can induce activation of IKKβ, leading to nuclear translocation of NF-κB and, as a result, the production of various inflammatory markers and potential mediators, and obesity can also co-promote phosphorylation of IRS1 at serine sites (ser302 and ser307) through JNK activation, which together leads to the development of IR (74). A majority of studies have found that inflammation plays a significant role in the development of IR, some other research has also shown that IR actually promotes the development of an inflammatory response. Both sides agree that inflammation and IR feed off each other in a vicious cycle (49). In both diet-induced obesity and genetically (mTORC2-knockout) induced adipose-specific IR, researchers discovered that IR causes local accumulation of pro-inflammation macrophages. IR produces the chemokine monocyte chemoattractant protein 1 (MCP1), which recruits monocytes and activates pro-inflammatory macrophages (75). Researchers discovered that blocking glucose oxidative metabolism not only prevented macrophage polarization to the M2 phenotype but also drove macrophages into the M1 type. Simultaneously, forcing an M1 macrophage to undergo oxidative metabolism boosts the M2 phenotype (76, 77). IR prevents glucose from entering neurons for oxidative phosphorylation, which explains the potential cause of IR-induced inflammation in terms of macrophage metabolic reprogramming.

### IR and oxidative stress

Oxidative stress and inflammation are two key points in ischemic stroke, and there has been considerable evidence in recent years that oxidative stress, associated with the overproduction of reactive oxygen species (ROS), is the underlying mechanism of brain injury in stroke (78). In ischemic stroke, excessive ROS production disrupts the balance of oxidant and antioxidant composition, resulting in oxidative stress (79). ROS are naturally occurring small molecule by products of oxygen metabolism that include superoxide anion radicals (O2^-^), hydrogen peroxide (H_2_O_2_), and hydroxyl radicals (-OH). The most reactive substance, -OH, can damage polyunsaturated fatty acids, causing loss of biofilm integrity, proteins, and enzymes, as well as affecting nucleic acids’ functional qualities and producing mutations that eventually result in cellular senescence or death (80). ROS are beneficial in certain physiological processes such as cell signaling, induction of pro-mitotic responses, immune defense, cellular senescence, apoptosis, and breakdown of toxic compounds. However, most studies indicate that ROS causes cellular damage and impaired function during biological stress (81). ROS can react with lipids to form peroxides, which are then degraded to aldehydes (e.g., hydroxynonenal) that are toxic to neurons and white matter in the brain, induce apoptosis, and are significantly associated with focal ischemia in rats (82). Increased ROS production after transient global ischemia upregulates p53 upregulated apoptosis regulator (PUMA) and Bcl-2 and Bax in neurons, and PUMA, along with anti-apoptotic Bcl-2 or pro-apoptotic Bax, plays an important role in ischemic neuronal death (83, 84). The effects of IR and oxidative stress also appear to be reciprocal. Oxidative stress-induced oxidative damage markers such as malondialdehyde (MDA), advanced glycation end products (AGEs), and 8‐hydroxy‐2′‐deoxyguanosine (8‐OH‐dG) were found to reduce insulin sensitivity in skeletal muscle cells and adipocytes (85). Some investigators believe that oxidative stress can phosphorylate IRS proteins by activating the IKKβ/NF-κB and JNK pathways, resulting in IRS degradation (86). Furthermore, some researchers believe that metabolic disturbances caused by peripheral IR may be the source of oxidative stress in brain tissue. IR increases FFA and promotes glucotoxicity and lipotoxicity and promotes NF-κB nuclear translocation to increase ROS production (87). Mitochondria are the main source of ROS, and in recent years, researchers have recently begun to study the relationship between mitochondrial function and IR. On the one side, researchers think in some cases, the reduced mitochondrial function may be a major cause of IR. They found that increased intracellular fatty acyl CoA and diglycerides in myocytes as a result of reduced β-oxidation due to mitochondrial dysfunction or increased plasma transport, activating serine/threonine kinases such as protein kinase C (PKC) in skeletal muscle. This, in turn, activates IRS1 serine residues, resulting in IR (88). Obesity is a common cause of IR. The 75-kDA glucose regulatory protein (GRP75) is downregulated in mice fed a high-fat diet (HFD); however, increasing GRP75 prevents HFD-induced obesity and IR. GRP75 is required for mitochondrial homeostasis because it is a component of the mitochondrial mass control system and mitochondrial-associated membranes. It can improve insulin sensitivity by regulating mitochondrial function by controlling the turnover of the mitochondrial-supercomplex (89). Furthermore, insulin is required for the maintenance of mitochondrial function. Researchers recently discovered that insulin deprivation not only resulted in decreased efficiency of ATP production by isolated muscle mitochondria but also increased degradation of mitochondrial proteins in streptozotocin (STZ)-induced diabetic mice treated continuously with insulin implants and mice that had their insulin implants removed after re-establishing healthy glycemic control. This increase in protein degradation could be attributed to increased expression of the mitochondrial autophagy marker protein (Beclin) (90). To better understand the connection between IR and mitochondrial function, researchers used IRS1 and IRS2 double-knockout mice to mimic IR. This study revealed increased expression of several forkhead box O1 (Foxo1) target genes in the liver of double knockout mice, including heme oxygenase-1 (Hmox1), which disrupts complexes III and IV in the respiratory chain and reduces the NAD+/NADH ratio and ATP production (91).

### IR and neuronal injury

Neurons are the most fundamental structural and functional elements of the nervous system. There is widespread agreement that prompt and effective neuroprotection following an ischemic stroke can significantly improve patients’ prognoses. In clinical practice, drugs (e.g., gangliosides) are widely used as neuroprotective agents in patients with ischemic stroke. However, the actual mechanism of neuronal death after ischemic stroke remains unknown. We discuss the association of IR with these mechanisms based on a recent review of the mechanisms of neuronal death after ischemic stroke (92). Ischemic stroke causes a large release of the neuroexcitatory transmitter glutamate, which leads to excessive activation of NMDA receptors and allows a large inward flow of calcium ions. Excessive intracellular calcium ion accumulation activates many calcium-dependent proteases, lipases, and deoxyribonucleases, leading to cell death. Moreover, calcium overload also causes the release of apoptotic factors from the mitochondria, which induces apoptosis (93). Excitotoxicity and inflammatory responses can cause not only receptor-interacting protein kinase 1 (RIPK1) activation to promote neuronal cell necrosis, but also DNA damage. Once DNA is damaged, p53 can promote ferroptosis by inhibiting SLC7A11 expression or promoting spermidine/spermine N1‐acetyltransferase 1(SAT1) and glutaminase 2 (GLS2) expression (94, 95). After an ischemic stroke, oxygen and glucose transport are restricted, anaerobic oxidative metabolism takes over as the primary source of neuronal ATP, and energy supply exceeds demand, resulting in neuronal apoptosis (96). oxygen-glucose deprivation also inhibits the AKT pathway leading to decreased mTORC1 activity, a classical pathway that blocks autophagy activation (97). Excessive ROS accumulation due to mitochondrial dysfunction activates FOXO3, increasing the abundance of LC3 to promote autophagosome generation, and excessive autophagy can increase neuronal apoptosis (98). Autophagy response dysfunction causes adipocyte dysfunction, and the development of IR. IR induces glucotoxicity, which exacerbates oxidative stress, inflammation, and endoplasmic reticulum stress caused by lipotoxicity, further impairing the autophagy response (99). Furthermore, our team found that IR inhibits GLUT4 membrane translocation, leading to the apoptosis of neurons due to insufficient glucose uptake. Inhibiting neuronal IR attenuates neuronal apoptosis (100). All of this suggests that IR could cause neuronal death. According to research, ischemic stroke patients’ declining memory and cognitive function may be caused by neural synaptic plasticity dysfunction (101, 102). Synaptic plasticity is an activity-dependent change in the strength of neuronal connections and has long been recognized as an important component of learning and memory (103). Considering that insulin is required for neurosynaptic function, IR also has an impact on neuronal function. It has the ability to alter synaptic plasticity in neurons (104). Neuronal injury may be responsible for poor clinical outcomes such as worsening neurological function and a poorer functional outcome at 3 months in patients with ischemic stroke due to IR. A new study discovered that maternal HFD-dependent IR impairs multigenerational synaptic plasticity, learning, and memory (105). By inducing IR in female mice with HFD and evaluating hippocampus-dependent synaptic plasticity and memory in female offspring (no difference between females and males), researchers found that offspring novelty recognition experimental preference indices were lower than controls and that only BDNF was reduced in all three generations of offspring mice. Epigenetic inhibition of exon-specific BDNF expression in offspring may explain how HFD-induced IR multi-generationally impairs synaptic plasticity, learning, and memory. Furthermore, maternal administration of BDNF or lack of pro-IR gene p66Shc abrogated the transmission of HFD-dependent cognitive dysfunction to offspring.

## How to treat IR in ischemic stroke patients

T2D is an established risk factor for ischemic stroke, however, numerous research has revealed that many ischemic stroke patients who do not have T2D but do have IR also have a significantly worse prognosis than those who do not have IR. Insulin is important for maintaining the functional integrity of the brain, and peripheral and central insulin dysfunction due to IR may be an independent risk factor for stroke. IR treatment is an important component of the secondary prevention of ischemic stroke (66). Increasing CNS insulin concentrations or CNS insulin sensitivity may be effective not only in preventing ischemic stroke but also in improving the prognosis of ischemic stroke patients. We discuss the treatment of IR in terms of non-pharmacological modalities, insulin therapy, and increasing insulin sensitivity (**Table 2**).

**Table 2:** The treatment of IR in ischemic stroke.

Therapeutic approach	Method	Mechanism
	Healthy diet	Improve insulin sensitivity
Non-pharmacological modalities	Smoking cessation	Improve insulin sensitivity
	Exercise	Improve insulin sensitivity
Intranasal insulin	Intranasal insulin	Activate AKT
	Thiazolidinediones	Activate PPAR-γ
	Metformin	Activate AMPK
Insulin sensitizers	Statins	Regulate lipid metabolism
	Astaxanthin, α-ketoglutarate	Inhibit mTOR/S6K1
GLP-1 receptor agonists	Semaglutide, Exendin-4, etc.	Active GLP-1 receptor

PPAR-γ, peroxisome proliferator-activated receptor γ; AMPK, AMP-activated protein kinase; GLP-1, glucagon-like peptide-1. IR, insulin resistance.

### Non-pharmacological modalities

Lifestyle changes, including a healthy diet, weight loss, smoking cessation, and appropriate physical activity are well-known ways to improve peripheral insulin sensitivity and are considered primary prevention of ischemic stroke (66). Obesity is associated with an increased prevalence of vascular risk factors, and obesity is typically the primary cause of IR. In the INTERSTROKE research, obesity was responsible for 82% and 90% of the population-attributable risk for ischemic and hemorrhagic stroke, respectively (106). The researchers discovered that a healthy vegetarian diet was substantially associated with a lower risk of overall stroke and that vegetarian diet was unrelated to stroke in a follow-up of 3,015 ischemic strokes and 853 hemorrhagic strokes (107). Compared with the control condition, folic acid was associated with a lower risk of stroke [relative risk (RR)= 0.80, confidence interval (CI)=0.67-0.96], whereas combined calcium plus vitamin D intake was associated with an increased risk (RR=1.17, CI=1.05-1.30) (108). Dietary control in diabetic patients is a simple, effective, safe, and efficient way to improve IR (109). Researchers identified smoking and exposure to secondhand smoke as definite risk factors for stroke in a comprehensive review of the Global Burden of Disease Study 2019 (110). Researchers found that both male and female smokers (cigarettes + e-cigarettes and cigarettes or e-cigarettes) were more likely to develop IR than non-smokers (male, dual: odds ratio (OR)=2.19, CI=1.39–3.44; single: OR=1.78, CI=1.43–2.22; female, dual: OR=2.32, CI=1.01–5.34; single: OR=1.76, CI=1.28–2.42) (111). There is sufficient evidence to incorporate cardiopulmonary and mixed training involving walking into post-stroke rehabilitation programs. Cardiopulmonary training, as well as, to a lesser extent, mixed training, improves movement and balance and thus reduces disability during or after conventional stroke treatment (112). Exercise and IR have a well-established link. IRS1 and GLUT4 mRNA levels were lower in the proliferating endometrium of patients with polycystic ovaries compared with BMI-matched controls. Lifestyle changes combined with diet and exercise resulted in improved menstrual patterns in 65% of overweight/obese women with polycystic ovary syndrome, and significantly higher IRS1 and GLUT1 mRNA levels were found in the endometrium of these women with improved menstrual function, suggesting that lifestyle interventions can improve insulin sensitivity in patients (113). Recent studies have consistently shown that moderate repetitive exercise for 30 minutes or longer at least three times per week for at least eight weeks improves insulin sensitivity in patients with diabetes, obesity, and metabolic syndrome. Improved insulin sensitivity may be associated with weight loss (114). In summary, lifestyle changes are not only an important part of the primary prevention of ischemic stroke, but they can also play an important role in the treatment of IR in the secondary prevention of ischemic stroke.

### Intranasal insulin

IR is essentially a decrease in insulin cellular utilization, and exogenous insulin supplementation is also an effective way to increase intracellular insulin concentrations. Insulin administration in stroke patients has shifted from intravenous to intranasal. In a meta-analysis including 9 studies (1,491 patients), there was no statistically significant difference in the incidence of mortality (OR=1.16, CI= 0.89-1.49) and improvement in neurological function (OR=1.01, CI=0.81-1.26) in patients treated with intravenous insulin compared to controls. However, the odds of any hypoglycemia (OR=8.19, CI=5.60-11.98) and symptomatic hypoglycemia (OR=6.15, CI=1.88-20.15) were significantly higher in patients receiving intravenous insulin therapy (115). The reason for this phenomenon may be that although peripheral insulin injections are the classical way to treat T2D, they may induce hypoglycemia in stroke patients with only an insulin-resistant state that has not yet developed T2D, and this hypoglycemia can cause secondary brain damage, and peripheral insulin injections may be ineffective due to impaired insulin transport by BBB. As a result, researchers have concentrated on intranasal insulin administration as a neuroprotective treatment for ischemic stroke. Intranasal administration is a safe and effective method of bypassing the BBB and increasing distribution to the CNS without the downsides of systemic side effects or first-pass metabolism while reducing IR (116). Investigators discovered that the incidence of vascular disease was not significantly higher in the insulin group than in the placebo group in a clinical study examining the safety of intranasal insulin administration for the treatment of Alzheimer disease dementia, also demonstrating that intranasal insulin administration is safe (117). Intranasal insulin administration reduced cerebral infarction and neurological deficits and increased phosphorylation of Akt and endothelial nitric oxide synthase (eNOS) proteins in STZ-induced diabetic rats with focal cerebral ischemia-reperfusion injury. When insulin is combined with N-iminoethyl-L-ornithine, an eNOS inhibitor, the beneficial effects of insulin on infarct volume and neurological deficits in ischemia-reperfusion diabetic rats are inhibited, but the hypoglycemic effect of insulin is not affected (118). Another hemorrhagic stroke study found that administering 1 IU of intranasal insulin significantly reduced hematoma volume, brain edema, and BBB permeability in intracerebral hemorrhage-induced mice. The researchers discovered that after intranasal insulin administration, the expression of AKT (Ser473) and GSK3 (Ser9) in perihematomal tissue was significantly increased. Insulin binding to its receptor activated AKT through serine phosphorylation at position 437, and the activated AKT promoted GSK3β phosphorylation at Ser9, which inactivated GSK3β. GSK3β was associated with neuronal death, and GSK3β over-activation induces neuronal degeneration and reduces the expression of claudin-1 and claudin-3, the major components of BBB tight junctions. The activation of the AKT/GSK3 signaling pathway by intranasal insulin may be the mechanism by which intranasal insulin administration improves neurological function in mice with cerebral hemorrhage (119). The evidence for intranasal insulin administration in protecting neurological function is sufficient, and intranasal insulin administration is a potential therapeutic modality for improving IR and restoring neurological function in ischemic stroke. In short, AKT is an essential pathway for insulin to exert its physiological effects, and although clinical trials of intranasal insulin administration for ischemic stroke need to be supplemented, the evidence for intranasal insulin administration in protecting neurological function is sufficient.

### Insulin sensitizers

Another viable strategy is to use insulin sensitizers to directly combat IR. Despite the fact that there have been few studies on insulin sensitizers in ischemic stroke, we have identified a number of useful insulin sensitizers based on previous research in T2D patients. Thiazolidinediones (TZDs) reduce insulin resistance directly by activating peroxisome proliferator-activated receptor γ (PPAR-γ), which promote mesenchymal stem cell differentiation into adipocytes, promote lipogenesis in peripheral adipocytes, lower hepatic and peripheral triglycerides, reduce visceral adipocyte activity, and enhance adiponectin (120). Two common TZDs are pioglitazone and rosiglitazone, both of which are PPAR-γ agonists. 3,876 patients who had recently experienced an ischemic stroke or TIA and had HOMA-IR > 3.0 but no T2D were split into two groups in a multicenter, double-blind experiment (pioglitazone or placebo). They discovered pioglitazone not only lowers the risk of T2D (hazard ratio (HR)=0.48, CI=0.33-0.69) but also the incidence of stroke and myocardial infarction (HR=0.76, CI=0.62-0.93) (121). 5,039 people with stroke or TIA participated in a meta-analysis of 5 RCTs, of which 4 evaluated the medication pioglitazone and 1 rosiglitazone (122). Additionally, it was discovered that PPAR-γ agonists, as opposed to a placebo, lower the incidence of recurrent stroke (RR=0.66, CI=0.44-0.99). Researchers discovered that PPAR-γ agonists decreased the composite outcome of major vascular events, such as cardiovascular death, non-fatal myocardial infarction, or non-fatal stroke total occurrences (RR=0.73, CI=0.54-0.99) in a single experiment with 984 patients. Since it doesn’t result in hypoglycemia, metformin, the most often used medication to treat T2D, is arguably the subject of the most research. Metformin exerts its activity through two main mechanisms: AMP-activated protein kinase (AMPK)-dependent and AMPK-independent modalities. When metformin activates AMPK, it can increase fatty acid oxidation by inhibiting the phosphorylation of acetyl coenzyme A carboxylase, ultimately improving lipid metabolism and insulin sensitivity (123). Metformin also has neuroprotective benefits in ischemic stroke by activating the AMPK pathway. Activated AMPK reduces neuroinflammation by inhibiting the release of inflammatory markers, decreasing ROS generation to reduce oxidative stress, and promoting autophagy. Furthermore, by reducing glutamate release, AMPK activation prevents glutamate excitotoxicity-induced apoptosis (124). Statins are lipid-lowering medications that are primarily used to treat cardiovascular disorders caused by high blood lipid levels, such as hyperlipidemia, hypertension, atherosclerotic heart disease, and others. Previous research has shown that long-term pravastatin plus captopril treatment improves the progression of IR and associated risk factors (hyperinsulinemia, hypercholesterolemia) (125). Another study came to the same conclusion, showing that in ob/ob mice—which developed obesity and IR due to a loss of functioning leptin, the infarct volume was considerably higher after middle cerebral artery occlusion (MCAO) than in wild-type or lean mice. Short-term treatment with rosuvastatin (10 mg/kg/day for 3 days) did not reduce infarct volume in wild-type and lean mice, but significantly reduced infarct volume in ob/ob mice with IR (126). However, ten weeks of intensive atorvastatin (40 mg/d) treatment increased the emergence of insulin resistance and insulin secretion in participants without diabetes (127). This conflicting outcome indicates that statins for insulin resistance still require additional experimental confirmation. Apart from this, several medicines (e.g., α-ketoglutarate, astaxanthin) can reduce IR and increase neuronal survival by decreasing aberrant serine phosphorylation of IRS1 directly *via* inhibiting mTOR/S6K1 (100, 128). In summary, there is substantial evidence that insulin sensitizers can boost brain insulin sensitivity, successfully treat ischemic stroke, and improve neurological function.

### GLP-1 receptor agonists

Glucagon-like peptide-1 (GLP-1) maintains glucose metabolism stability by increasing insulin production and suppressing glucagon secretion, enhancing cell proliferation and growth, and avoiding cell excitotoxicity and death (129). GLP-1 receptors (GLP-1Rs) are found throughout the CNS (130). GLP-1 receptor agonists (GLP-1RAs) not only be used to treat T2D by increasing peripheral insulin sensitivity but have also been found to increase neuronal sensitivity to insulin (131, 132). In a meta-analysis of 33,457 subjects, GLP-1RAs treatment was found to reduce the incidence of major adverse cardiovascular events (cardiovascular death, non-fatal myocardial infarction, and non-fatal stroke) compared to placebo (HR= 0.90, CI=0.82-0.99) and did not cause adverse effects such as severe hypoglycemia (133). Another meta-analysis, which included 35 preclinical studies, 11 retrospective database studies, 7 cardiovascular outcomes trials, and 4 prospective clinical studies, found that administration of GLP-1RAs after stroke reduced infarct volume, apoptosis, inflammatory responses, and oxidative stress, while also promoting neurogenesis, angiogenesis, and increased cerebral blood flow to exert neuroprotective effects. Furthermore, in cardiovascular outcome trials, dulaglutide and semaglutide were observed to minimize the risk of stroke (134). Exendin-4, another GLP-1RAs, reduces the activation of astrocyte-derived matrix metalloproteinase-9 (MMP-9), vascular endothelial growth factor (VEGF-A), MCP1, and chemokine C-X-C motif ligand 1 (CXCL-1) by oxygen-glucose deprivation (OGD), as well as the activation of the JAK2/STAT3 signaling pathway by OGD. Exendin-4 dramatically improved neurological function scores and reduced infarct volume in MCAO rats, yielding similar outcomes (135). Taken together, GLP-1RAs are a very promising medicine for the treatment of IR in ischemic stroke, but additional clinical trial evidence is required.

## Conclusions and perspectives

**Figure 3:**
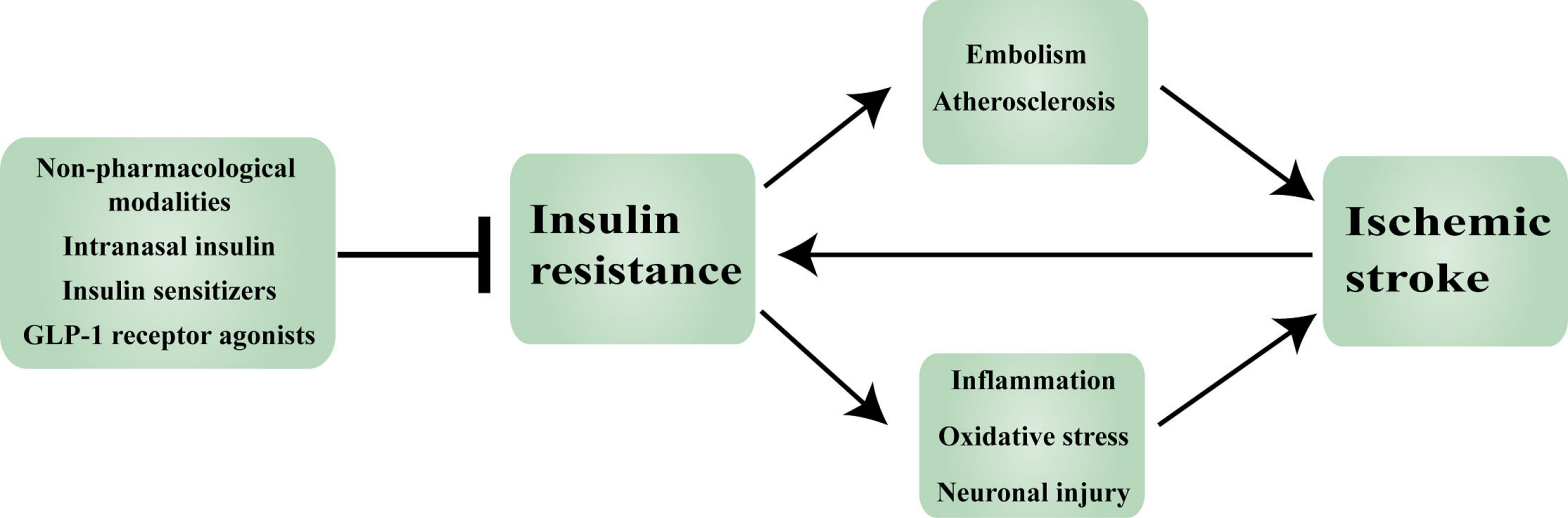
Insulin resistance in ischemic stroke: mechanisms and therapeutic approaches. GLP-1: Glucagon-like peptide-1.

Insulin is essential for brain function. IR, which leads to insulin dysregulation, can cause neurological damage *via* a variety of mechanisms. Numerous studies have shown that IR is not just a feature of T2D, but it also plays a key role in the development and progression of ischemic stroke. IR is an independent risk factor for ischemic stroke, IR contributes to the development of ischemic stroke by promoting thrombosis and atherosclerosis formation. IR, which is associated with a poor prognosis in ischemic stroke patients, exacerbates the inflammatory response, oxidative stress, and neuronal damage. Current IR treatment in patients with ischemic stroke is based on medicine selection based on T2D treatment experiences, such as insulin, insulin sensitizers, and GLP-1RAs. In many studies, insulin sensitizers and GLP-1RAs had no major side effects in patients with ischemic stroke, however, the use of insulin has attracted controversy. Although peripheral insulin injection therapy is the preferred treatment for T2D, it has a number of negative effects in ischemic stroke patients, and safer and more effective intranasal insulin administration is becoming more common (**Figure 3**). However, these studies still have some limitations, the first of which is the dispute over how to identify IR. For instance, while some authors consider HOMA-IR >3 to validate the diagnosis of IR, others define IR as HOMA-IR ≥2.5. Although ethnicity may be a factor in the cut-off value heterogeneity, a consistent scoring standard can boost the credibility of experimental findings. Secondly, further research is needed to fully understand the variety of roles that IR plays in ischemic stroke. Aside from the inhibitory effects of insulin on the regulation of glucose metabolism, inflammatory response, oxidative stress, neuronal function, and vascular function, there could be additional mechanisms that modulate ischemic stroke that is independent of the insulin signaling pathway. Overall, there is mounting proof that IR is an independent risk factor for ischemic stroke and a major component in poor patient prognosis. The prevention and treatment of ischemic stroke may benefit from a systematic understanding of the mechanisms behind IR in ischemic stroke.

## Author contributions

P-FD wrote the manuscript. H-SZ, JW, C-HH and WL contributions to design of the work. Y-YG and J-NM made suggestions for some of the work. C-HH and WL revised the work. All authors contributed to the article and approved the submitted version.
